# Transformative Encounters: A Narrative Review of Involving People Living With HIV/AIDS in Public Health Teaching

**DOI:** 10.3389/phrs.2022.1604570

**Published:** 2022-06-22

**Authors:** Yudit Namer, Florian Drüke, Oliver Razum

**Affiliations:** ^1^ Department of Epidemiology and International Public Health, School of Public Health, Bielefeld University, Bielefeld, Germany; ^2^ Research Institute Social Cohesion, Bielefeld, Germany

**Keywords:** narrative review, public health education, HIV/AIDS, patient involvement, PLWHA

## Abstract

**Objectives:** To collate the experiences of involvement of people living with HIV/AIDS (PLWHA) in academic public health teaching to inform future public health education models involving people affected by long-term effects of other pandemics. Our goal is to describe interventions in a way that makes them accessible to potential public health teachers hoping to adapt patient involvement paradigms in their teaching of chronic illness brought on by infectious diseases.

**Methods:** Narrative review based on a literature search in PubMed and Google Scholar up to September 2021. Fifteen articles that contained a description of a health educational intervention on HIV/AIDS that actively involved PLWHA were included.

**Results:** Interventions either involved PLWHA as teachers and program/curriculum developers or incorporated experiential elements in which students have genuine contact with PLWHA. Creating safe spaces, recognizing PLWHA as experts, relating to each other differently were common transformative elements.

**Conclusion:** Involving PLWHA in public health teaching have transformative and empowering outcomes, both for PLWHA and for learners. This finding should inform new teaching programs that will address the long-term effects of other pandemics such as COVID-19.

## Introduction

For many communities, the COVID-19 pandemic has been the largest and most intrusive health emergency that they encountered. It was the first time that their social relationships have been subjected to rules and regulations, the distance between each other has been surveilled, and forms of social contact became a threat. For many, it was the first time they lost friends and family members on such a scale. However, none of this was new for communities whose lives were impacted by the HIV pandemic, which has been with us for 40 years, affecting an estimated 38 million people world-wide (1).

The similarities and differences between the HIV and COVID-19 pandemics are increasingly becoming part of the scientific discourse. Although the routes of transmission are different, the disproportionality of marginalized communities being affected, the stigma-related barriers to social participation, the necessity of stigma reduction to tackle misinformation and to promote prevention are argued to be the similarities between the two pandemics (2–4). Moreover, the initial denial of the severity of both infectious diseases by some scientists, health care professionals and affected communities alike, shadowed by stark panic by the public that targeted certain communities followed a similar narrative in both pandemics (5). Patient activism is another similarity: both in the cases of living with HIV and living with Long COVID there were (and still are) “epistemic authority” struggles, with especially people from marginalized communities fighting to get their lived experience recognized as expert knowledge (6).

Such discussions surrounding pandemic preparedness in response to COVID-19 made it apparent that communities’ knowledge of surviving HIV/AIDS has not been adopted by the larger society (7) and is underutilized by scientists and health professionals (8). We believe that the lack of knowledge transfer also applies to the experiences of community involvement in medical and public health teaching. In other words, paradigms of patient involvement have not been adopted widely enough to allow future health professionals the opportunity to learn from lived experience.

### Patient Involvement in Teaching

Patient involvement, or inclusion of people with lived experience of illness in teaching is a component of knowledge transfer that adds significant value to medical and public health education. Patients are experts in their own experience of living with certain health issues (9). They provide expert advice regarding symptom management or coping with the consequences of ill health. They also demonstrate that health problems manifest in unique and heterogeneous ways (10). Patients embody the social determinants of health and illustrate a larger picture of living with a health condition, especially in the cases of chronic illness (11). Contacts with patients thus challenge long-held attitudes and dispel myths about health conditions (12, 13). Despite its value being recognized, there is yet to be wide and systematic adoption of patient involvement in medical and public health teaching (14).

Towle and Godolphin (15) identified three guiding principles of patient involvement in teaching. First, patients possess knowledge by experience that cannot be taught by faculty. Second, power relations should be balanced to ensure authentic participation of patients. Third, patients should have the say in what, how and where they teach. Towle et al. (11) further developed levels of involvement from minimal involvement to high: 1) patient as case study or scenario, 2) patient as volunteer in a scripted clinical encounter, 3) patient sharing experience in faculty-shaped framework, 4) patient assuming the role of teacher or evaluator of student performance, 5) patient as equal partner in curriculum development, and 6) patient holding sustainable institutional membership. A recent review of patient involvement in undergraduate medical education found that patients most commonly take the role of teacher, and with slightly less frequency the roles of assessor, curriculum developer, and student selection committee member in active involvement paradigms (14). In other words, interventions increasingly adopt elements of levels 4 to 6 of Towle et al. framework.

Patient involvement in teaching can be based on different principles depending on the health condition. The Greater Involvement of People Living with HIV (GIPA) is a “principle that aims to realize the rights and responsibilities of people living with HIV, including their right to self-determination and participation in decision-making processes that affect their lives” (16). PLWHA should therefore be included in the educative processes that go into building a health care workforce providing respectful, equitable, dignified health care. Although the GIPA as well as the practices and pedagogies of patient involvement in teaching have existed for decades and were adapted to the teaching of the HIV pandemic, they have not become core components of medical and public health teaching. PLWHA have identified “weak management, low skill levels, funding constraints, difficulties in representing the diversity of people living with HIV, a lack of documentation of their histories of self-empowerment and a lack of evaluation of successes and failures” as barriers to upholding the GIPA (16). It is possible to argue that similar reasons have obstructed the meaningful involvement of PLWHA in public health teaching.

The aim of this review is therefore to collate the experiences of involvement of PLWHA in teaching to inform future public health education models involving people affected by long-term effects of other pandemics. Our goal is to describe interventions in a way that make them accessible to potential public health teachers hoping to employ patient involvement models in their teaching of chronic illness brought on by infectious disease. We present different forms of involving PLWHA in teaching and assemble the lessons learned from these interventions. We then discuss the possibility of patient involvement for a resilient and transformative public health education post-COVID.

## Methods

To identify studies for this narrative review we performed a literature search in PubMed and Google Scholar including papers published up to September 2021 using variations and combinations of the search terms: HIV, AIDS, medical education, public health education, continuing education, professional development, community, resilience, survivorship and involvement. The search strategy was developed through an iterative process by trying different permutations of search terms. There were no restrictions in terms of year of publication, but the search language was limited to English. Our final search in PubMed with the following keywords revealed 153 results:

(“HIV”[Title/Abstract] OR “AIDS”[Title/Abstract] OR “HIV/AIDS”[Title/Abstract]) AND (“medical educa-tion”[Title/Abstract] OR “public health education”[Title/Abstract] OR “continuing education”[Title/Abstract] OR “professional development”[Title/Abstract] OR “medical curriculum”[Title/Abstract]) AND (“community”[Title/Abstract] OR “survivor”[Title/Abstract] OR “resilie-nce”[Title/Abstract] OR “involvement”[Title/Abstract])

All peer-reviewed publications were screened by title and abstract by YN and FD in the first round and by full text in the second round according to the inclusion and exclusion criteria presented in Table 1.

**TABLE 1:** Inclusion and exclusion criteria for the literature search.

Inclusion criteria	Exclusion criteria
• The article included a description of a health educational intervention on HIV/AIDS.	• The experience of living with HIV/AIDS is not central (e.g., only used as an example).
• The education intervention actively involved PLWHA.	• The education intervention is not in the health sciences.
• The article was published in a peer-reviewed journal.	• Grey literature or non-peer-reviewed scientific publications.

**FIGURE 1:**
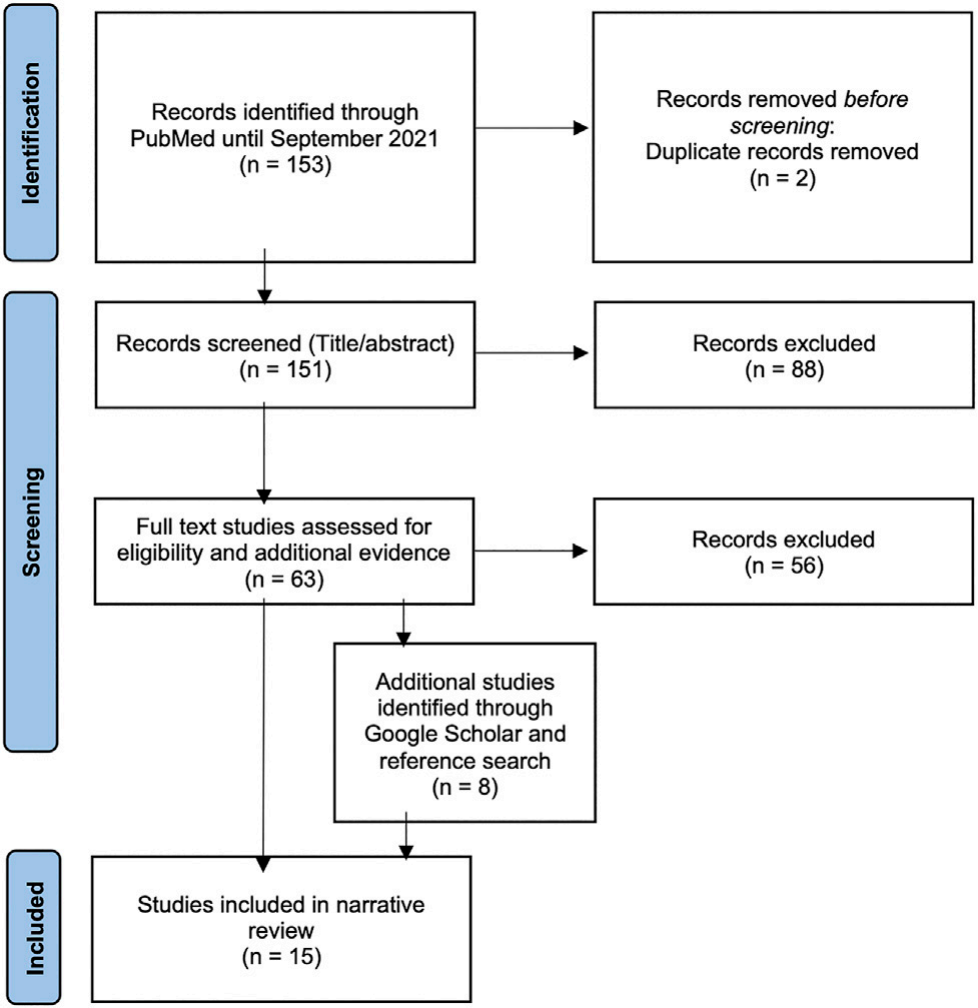
Identification of studies for the narrative review (Bielefeld, Germany. 2021).

Additional studies were retrieved by screening the references within the relevant articles by YN and FD. Although our aim is to draw conclusions for public health education, we included literature on medical, nursing and other health education as well to capture the few published experiences of PLWHA involvement. A total of 15 articles were included in the review. The selection process is displayed in Figure 1 (adapted from (17)).

## Results

In response to the emergence of HIV/AIDS, the first articles discussing the teaching of HIV date from the early 1990s. These studies were a response to the myths surrounding the then-AIDS epidemic and the fears and reservations they instilled. More teaching interventions seem to have emerged in the second decade of the pandemic, with a decline in the third decade, and another increase in the fourth decade.

We discuss the interventions in detail to give a vivid picture of how PLWHA were involved in teaching. We organize the results in three categories: incorporating experiential elements in which students have genuine contact with PLWHA (level 3 of involvement) or interventions involving PLWHA as teachers/instructors/educators (level 4 of involvement) and as program/curriculum developers (level 5 of involvement) (11). An overview of findings is presented in Table 2.

**TABLE 2:** Overview of findings.

Level of involvement (Towle et al. 2010)	Study	PLWHA involvement in teaching intervention	Learners	Evaluation
Level 3	(18, 19)	Participants (1) attended panel presentation by a support group for PLWHA, (2) attended support groups, and (3) joined community network meetings of HIV health care providers	Community mental health professionals	Learners: increase in knowledge, and decrease in fear, discomfort and avoidance related to HIV
Level 3	(20)	Participants attended theatrical performance based on the performers’ lived experience of HIV	Medical students and physician faculty	PLWHA: felt their experiences mirrored
				Learners: gained new insights about the PLWHA perspective, seeing a PLWHA as a full person rather than reduced to a diagnosis
Level 4	(21)	Experiential sessions in which PLWHA told their stories and shared their clinical skills	Medical students	PLWHA: empowered in their roles as teachers
				Learners: improved their relationships with their patients and made them more open to discuss their own fears and anxieties
Level 4	(22, 23)	PLWHA as “resource tutors” facilitating reflective spaces	Nursing, social work, occupational therapy, physiotherapy students and family medicine residents	PLWHA: empowered, discovered new aspects of self, found purpose in teaching, change in experience of HIV
				Learners: confronted their assumptions about HIV, accepted PLWHA as knowledge sources into their experience, learning new ways of communication
Level 4	(24)	PLWHA facilitated body mapping workshops	Nursing students	Collective: connection, trust, commitment to fight stigma and demand social justice for shared lives
				PLWHA: validation of experience, giving back to community
				Learner: change in attitudes, moved from initial ignorance to future commitment to HIV advocacy
Level 5	(12, 26)	PLWHA formally employed as primary faculty developing the teaching materials on HIV/AIDS as well as assuming the teacher role	Medical students/trainees, health care professionals (e.g., nurses, social workers, dentists, physicians, psychologists)	PLWHA faculty: change in motivation over time, from personal reasons (e.g., financial) to relational reasons (e.g., effecting change for others), empowered, took agency in their HIV experience, found a support network within the program
				Learners: focus on the similarities of PLWHA and their own lives
Level 5	(27–29)	PLWHA co-developed program and facilitated discussions on their lived experience and co-created a simulated clinical encounter (SCE) intervention and delivered it as patient instructors	Medical students	PLWHA: empowered, felt respected as experts, found a safe space to relive experiences
				Learners: reduction in HIV-related stigma, increase in the willingness to deliver HIV care
Level 5	(30)	PLWHA co-created a narrative medicine curriculum	Medical students	Collective: relationships based on reciprocity and a sense of community
				PLWHA: felt their contribution to medical education meaningful
				Learners: found meaning in contributing to the PLWHA’s wellbeing
(elements of) Level 5	(32)	Consultation in curriculum development	Community-based paraprofessionals	—
(elements of) Level 5	(33)	PLWHA engaged in photo elicitation and photovoice to create materials for teaching purposes	Dental care students and professionals	—

### Experiential Modules Incorporating Genuine Contact With PLWHA (Level 3)

The earlier teaching interventions created opportunities for students to have contact with PLWHA in a meaningful way. The first study that incorporated organic contact with PLWHA into teaching came from the first decade of the epidemic and the field of community mental health (18). Following an introduction on the relationship between community mental health and living with HIV/AIDS, and clinical and epidemiological information on HIV/AIDS, Knox et al. introduced experiential components involving PLWHA in clinical tutorials in three modalities. First, the tutorial participants attended a panel presentation by a support group for PLWHA with mental health-related histories. Second, they attended the support groups themselves, either aimed at people living with HIV, people living with AIDS, or their loved ones. Third, they joined community network meetings of HIV health care providers and agencies prioritizing mutual support. A subsequent quantitative evaluation of this clinical tutorial demonstrated increases in knowledge, and decreases in fear, discomfort and avoidance related to HIV (19).

The next study involved the PLWHA perspective within an artistic framework (20). In this study by Shapiro and Hunt, medical students attended an hour-long theatrical performance based on the performer’s lived experience of HIV as part of a course module. The performance involved storytelling through songs the performer wrote about his life as a PLWHA. The audience was heterogenous, made up of physicians, students, other PLWHA, their family members and community health workers. The performance was followed by a panel discussion of physicians, scholars and the performer with the audience and then an informal gathering. In an informal evaluation the attendees reported different reactions. PLWHA audience members felt their experiences mirrored. Physician faculty stated identifying with the patient in the performance and thus gaining new insights about the PLWHA perspective. The student attendees appreciated learning about the HIV experience without the element of providing patient care. Students further favored watching how doctors were portrayed in the performance and seeing a PLWHA as a full person rather than reduced to a diagnosis.

### Inviting PLWHA as Teachers (Level 4)

The first study we found that exemplified inviting PLWHA as teachers was by Vail et al. (21). The intervention invited support group-attending PLWHA (who therefore have the experience of sharing their stories) to join a minicourse for medical students. The preparation for PLWHA comprised a discussion on the clinical skills and attitudes required to care for PLWHA and a workshop with a medical student to practice giving feedback to students. The resulting minicourse incorporated lectures on the epidemiology of HIV by faculty followed by experiential sessions in which PLWHA told their stories. The narratives spanned from disclosures to loved ones to anxieties and fears about dying. In the next phase, PLWHA participants were paired with students, practiced clinical skills one-on-one and provided feedback on students’ performance. The minicourse evaluations revealed that this involvement benefited both parties immensely: PLWHA felt empowered in their roles as teachers and students were deeply and positively changed by the experience in a way that improved their relationships with their patients and made them more open to discuss their own fears and anxieties.

A second group of studies by Solomon et al. involved PLWHA as “resource tutors” in small-group problem-based tutorials (22, 23). This model recruited PLWHA who either already had experience working with small groups (22) or received relevant training to do so (23). Faculty tutors undertook the facilitation of tutorial sessions while the resource tutors created the reflective spaces for the students to discuss different care-related problems. There were also opportunities for role-playing care scenarios with resource tutors (22, 23). In the qualitative evaluation based on reflective journal entries and interviews, it was apparent that the tutorial encounters allowed the students to confront their assumptions about HIV and accept PLWHA as knowledge sources into their experience (22). Resource tutors were already highly motivated to help future health care professionals, to learn how to teach and to discover new parts of themselves when they joined the teaching project. Some reported initial uneasiness with sharing their experiences. As the sessions progressed, however, resource tutors gained more confidence and adapted their teaching styles to incorporate more creative techniques to convey their lived experience (23).

A third study involved PLWHA in facilitating body mapping workshops in collaboration with a community HIV organization (24). The body mapping method emerged out of community work with PLWHA and comprises creating large body-size paintings that tell the story of the painter’s physical as well socio-emotional experience of living with HIV (25). In this study, Maina et al. used it as part of a community nursing course. PLWHA facilitators led the workshops in which the students created group body maps and observed the body maps of PLWHA. A post-mapping session held with the course professor, a PLWHA facilitator, a community organization facilitator and a student highlighted the importance of being vulnerable in the teaching space, and the responsibility of the course facilitators to deem the teaching space safe. It also demonstrated how sharing one’s bodily vulnerabilities and life stories allowed people to connect to and trust each other. The validation of each other’s stories further generated the commitment to fight stigma and demand social justice for their shared lives. The exercise was especially transformative for the student, who reported moving from initial ignorance to future commitment to HIV advocacy through the workshop.

### Inviting PLWHA as Program/Curriculum Developers (Level 5)

The first group of work that described an approach involving patients as comprehensive program developers also critically reflected on the experience. The outcome thus became seminal papers on working with PLWHA as faculty members. Hatem et al. (26) expanded on Vail et al.’s model (21) and formally employed PLWHA as primary faculty developing the teaching materials on HIV/AIDS as well as assuming the teacher role. The preparation was extensive and cumulative: PLWHA faculty first audited a program, and once recruited received orientation and attended programs taught by fellow PLWHA faculty. Formal training in program development and teaching followed the decision to commit to the program. The co-developed programs included panel discussions, small group teaching or small group discussion facilitation. PLWHA faculty were debriefed, regularly monitored for burnout and checked up on during their time off by non-PLWHA faculty (26). A report on the learners’ experience of an earlier version of this teaching approach showed that involving PLWHA allowed learners to focus on the similarities of PLWHA and their own lives (12). A qualitative evaluation with the PLWHA faculty revealed a change in motivation over time, from personal reasons (e.g., financial) to relational reasons (e.g., effecting change for others). The PLWHA faculty reported feelings of empowerment, a sense of mutual understanding and taking agency in their HIV experience (26).

The second group of work involved PLWHA in curriculum development in a participatory way. In the first study, Chew et al., most of whom were medical students at the time, consulted with PLWHA and community HIV organizations in addition to medical students and HIV care physicians to develop a medical student-initiated extracurricular HIV elective course (27). The course incorporated discussion sessions led by PLWHA on their lived experience of HIV and visits to community care organizations. Building on their previous work, the team further co-created a simulated clinical encounter (SCE) intervention together with PLWHA who then delivered it as patient instructors (28, 29). The intervention involved HIV testing and pre- and post-test counseling encounters with two scenarios: a negative and a positive test result. The PLWHA instructors acted as the patient in SCEs with medical students, provided the majority of the feedback in the post-SCE sessions and were available for questions. The evaluations revealed that the PLWHA found their role in medical training meaningful and empowering. They felt that the intervention was a safe space where they felt respected and seen as experts. Both groups thought the intervention was realistic and feasible, and contributed to student skill-building (28). The intervention also reduced HIV-related stigma in medical students and increased the willingness to deliver HIV care (29).

One other participatory study involved a narrative medicine curriculum (30). Narrative medicine as a model examines different narrative situations in medicine and incorporates narrative practices such as close reading of texts and reflexive writing (31). In this “community-based participatory narrative medicine” study, Chou et al. involved preclinical medical students and patients receiving care in a clinic which typically serves PLWHA, although they did not require participants to reveal their HIV status (30). The intervention contained participants’ narrative writing which happened outside of sessions, close reading of texts selected by the facilitators based on participants’ diverse backgrounds, in-session writing based on prompts, and workshopping participants’ writing. Evaluations shaped by observations, participant feedback, focus groups and the narratives themselves showed that this intervention allowed participants to build relationships based on reciprocity and a sense of community. Both groups found a safe space where they helped each other reflect on their own and each other’s lives by sharing their writing. PLWHA felt their contribution to medical education meaningful and medical students similarly found meaning in contributing to the PLWHA’s wellbeing. Both groups remarked that the relationships they built through this intervention were unlike typical patient-medical student relationship.

Two studies further involved PLWHA in teaching material development in various ways. In developing a program aimed at community-based paraprofessionals, Poindexter et al. held focus groups with stakeholders, including PLWHA who have received services, to identify what skills people working in the field of HIV should possess (32). Although PLWHA were not involved in the drafting of the curriculum per se, they were consulted in the development process. Another study involved PLWHA more intensely in teaching material development for dental care students and professionals (33). In a grounded-theory action research, Schrader et al. invited PLWHA to engage in photo elicitation and photovoice and to keep reflective journals in order to visually represent their lived experiences. By creating photographs of important places or people in their lives, different community-oriented action themes emerged to contextualize PLWHA’s daily lives, such as recovery tools, social support and medications. At the end of the study, researchers created multimedia narrative posters for each meta-theme featuring the participants’ photos to be used for teaching purposes.

## Discussion

This narrative review collated experiences of involving PLWHA in teaching at various levels of involvement. The evaluations, taken together, suggest that involvement of PLWHA leads to a meaningful encounter of teaching and learning. We first discuss the elements that were transformative for both PLWHA and learners and the challenges of involving PLWHA in teaching. Following the limitations of this review, we discuss how these insights could be utilized in involving people with other chronic infectious diseases and what this could mean for the future of public health education.

### Transformative Elements

Most of the papers we reviewed reflected on the intervention as a whole and shared the lessons they learned through running or facilitating the intervention. It was evident that the authors were inspired by creating a space for PLWHA and students to have a meaningful connection. The importance of creating a safe space before, during or following the teaching of the course was continuously underlined (21, 24, 28, 30). Some education interventions worked well because the PLWHA participants belonged to the same support group, which was a chosen family substitute for many PLWHA at the time (18, 19, 21). Support group members already possessed the experience of self-reflection as well as creating a reflective space for each other. Empowerment of PLWHA through meaningful contribution to education and being recognized as experts was a common and key transformative thread in most of the interventions (21–23, 26, 28). PLWHA felt empowered in their roles as teachers. Learners were deeply and positively changed by the experience in a way that improved their relationships with their (potential) patients and made them more open to discuss their own fears and anxieties.

Some education interventions were believed to be successful as the duration of the program allowed the PLWHA and students to form a meaningful relationship (21, 22, 30). Sustainability was further critical: some interventions expanded in content and scope and even increased the degree of participation as they were allowed to progress (19, 23, 27). The theatrical intervention was argued to be transformative due to the heterogeneity of the audience, the informality of the post-performance gathering and the liveness of the intervention, which increased the richness of the experience for the students (20). The interventions’ focus on the similarity between people was a significant transformative element (12, 20, 24, 30). Close bonds between participants were achieved through shared work, lack of salience of patient/medical roles in the intervention and a joint reimagination of the health care relationship (21, 24, 30). Experiencing a different way of relating to each other was another common transformative element (21, 24, 28, 30). Learners had an enhanced experience when the goals were clear for everyone involved, and when there was space and time to process their learning experience (12).

### Challenges

Although transformative, involving PLWHA brought with it a number of challenges. In the earlier years of the HIV pandemic, being open about HIV status meant loss of other employment for some PLWHA faculty (26). Their role exposed them to others’ negative attitudes (22, 23, 26). The PLWHA faculty’s role further necessitated negotiating their own mortality, grief and that of fellow faculty (21, 26). In the studies conducted relatively early in the HIV/AIDS pandemic, before the development of more effective therapies, the PLWHA faculty became ill with time. Involving PLWHA faculty then also meant making space for death and mortality-related issues. Progression of illness impacted teaching styles and created differences of interpretation of teaching approaches between PLWHA and non-PLWHA faculty (26).

Negotiating negative emotions was another challenge (22–24, 28). The simulated clinical encounter, for example, triggered negative memories of the PLWHA instructors’ own test encounter. Some studies further reflected on the time and energy required of the PLWHA facilitators to create a safe space for participants to explore participants’ vulnerabilities while their own vulnerabilities are also exposed (24). Yet the risk of re-traumatization by revisiting past experiences also came with the possibility of healing (24, 28). Challenges included the logistical issues of attracting audience members or participants to an extracurricular event. Scheduling was a difficulty in some studies: medical students’ schedules did not always allow participation in extracurricular events (20) and a similar problem ensued with community schedules in organizing community-based events (27). Communication was also reported as a challenge. In one group of studies, the students found it difficult to use the correct terminology in referring to the PLWHA experience, whereas PLWHA had difficulty with medical terminology used in the sessions which focused on parts of people rather than people as a whole (22, 23).

Several recommendations were made to remedy these challenges. Accepting the PLWHA experience as primary in differences of interpretation was a core recommendation (26). For logistical challenges, suggestions included partnering with student and community organizations to ensure attendance (20, 27). In the case of re-traumatization risk, training, debriefing as well as the availability of an on-site counselor were recommended (28). Other challenges also elicited the recommendation of additional training for PLWHA tutors in the cases where training would indicate overcoming of role-related anxieties (23). This recommendation came with a dilemma: as PLWHA were considered experts in their experience they weren’t always provided with additional training in HIV. However sometimes this created the risk of students evaluating the tutorials as somewhat incomplete (23). Clear communication of initial goals were argued to prevent some of the training-related challenges (12, 23, 26).

### Limitations of This Review

A core limitation of this review is the scope of the literature surveyed. We performed searches only on PubMed and Google Scholar and scanned and selected peer-reviewed journal articles. We realize that many such teaching interventions may not have been published in scientific journals but rather described in faculty newsletters, society meetings, or reports by community organizations. It is also possible that interventions were developed as part of graduate work, outlined in theses or dissertations, or in non-academic settings such as civil society initiatives. We acknowledge that we only reviewed part of the knowledge produced in PLWHA involvement in teaching. We further recognize that a narrative review is a less systematic approach to reviewing the available evidence. However, we provide a snapshot of what knowledge is immediately available to academic faculty members within knowledge sources accessible to future public health teachers.

### Recommendations and Implications for a Transformative Public Health Education

The results of this narrative review have several implications for the future of public health education. The aftermath of COVID-19 will have significant ramifications in terms of new chronic illnesses on a global scale and there is an increasing need to understand the lived experiences of new chronic conditions such as Long COVID. Although principles of involvement are yet to be developed for people living with Long COVID, many patient groups, including doctors who experience Long COVID, have published calls for recognition (34–36). These calls range from data generation on persisting symptoms of COVID-19 (“count Long COVID”) to acknowledging the experiences of people who managed their symptoms in isolation without a diagnosis but now need rehabilitative care. The common thread is the demand of patient involvement in policy and research. Based on the experience on involving PLWHA in teaching, we extend the call to involve people living with Long COVID and other potential chronic illnesses in public health education.

First of all, people with persisting post-infection symptoms should be recognized as experts and actively involved in public health teaching, evaluation and curriculum development, with the aspiration of sustainable membership in schools of public health, with equitable compensation. The latter could take the form of building permanent yet dynamic community advisory boards which inform student selection, program evaluation and educational agenda setting. Second, people with lived experience assuming such roles should be fully supported to prevent re-traumatization or burnout triggered by sharing or re-living of their experiences. This would necessitate creating equitable and safe spaces, opportunities to reflect on the teaching experience, monitoring for burnout and securing paid leave when signs of burnout are evident. Third, the focus should be on relational aspects of teaching. This means that sufficient time is allowed for students, faculty members with and without lived experience to spend time together in formal and informal settings to get to learn from each other. Fourth, when challenges, disagreements or differences in interpretation arise, the lived experience of living with chronic conditions should be recognized as primary.

In conclusion, the decades-long yet sparse experience of involving PLWHA should be recollected to inform the new teaching initiatives that parallel the emergence of new chronic conditions. Involving PLWHA has been shown to be transformative and empowering for all parties, with unique challenges spanning from logistical to relational. Future studies should explore the exact means necessary (e.g., management, funding, pedagogical training) to ensure sustainability of involving lived experience in a transformative way.
